# The Successful Use of Colchicum in Hysteria and Hypochondriasis

**Published:** 1817-01

**Authors:** Thomas Raven

**Affiliations:** House Pupil to the Norfolk and Norwich Hospital.


### *For the London Medical and Physical Journal.*

*The successful Use of Colchicum in Hysteria and Hypochondriasis.* Communicated by Mr. THOMAS RAVEN, House Pupil to the Norfolk and Norwich Hospital.

ANN HOWARD, æt. 21, was thrown into strong convulsions by seeing a relation in the agonies of death. As these convulsions returned every day, and with considerable violence, the patient was entered at the Dispensary of this city; and, I understand, went through a regular course of fœtids, volatiles, cathartics, and tonics. But, as these medicines afforded her no relief, she made application to our hospital, and was admitted under the care of Dr. Alderson, who, having lately seen, in seven cases of chorea, the good effects of the meadow saffron, was induced to try the virtue of it in this attack of hysteria. Thirty drops of the tincture were ordered to be taken every eight hours. The effect of the medicine in this case was as surprising as it had been in the chorea, for in a few days the convulsions left her, and have not returned since.

A man from the country, between thirty and forty years of age, apparently in high health, had wearied two practitioners in his neighbourhood, and all his acquaintance, by a doleful detail of his very miserable feelings. Being thus a burthen to others, as well as a torment to himself, he was sent to our hospital, and admitted by Dr. Alderson, but rather reluctantly, because his disorder appeared of a kind that could not be removed in the six weeks allowed by our rules and orders for a trial. At the period mentioned, he thought himself worse than when admitted; he had taken a variety of medicines, and the doctor would have put him upon the list of incurables, but that he wished to see what effect the colchicum would have upon this disease, and prescribed as follows: Bibat vini colchici autumn. cochlear. parv. i. 8vis horis augendo vel minuendo dos. prout res postulet. As the first three spoonfuls operated very powerfully on the stomach and bowels, I lessened the dose one-half, and the medicine afterwards seemed inert. In ten days, however, after he had began the drops, he said they had done him more good than all the other medicines he had taken, that he was quite a new man, and was sure, if he went on with them a fortnight or three weeks longer, he should be well. His prediction proved true; he was discharged cured, but begged to be left on the list of out-patients, that, in case his disorder returned, he might send for a bottle of the tincture, which had done him so much good.

Without

Without any other comments on the cases which I send you, I may be allowed to say, that they are related with the strictest regard to truth.

*Norwich; December* 6, 1816.

